# Frontiers of ferroptosis research: An analysis from the top 100 most influential articles in the field

**DOI:** 10.3389/fonc.2022.948389

**Published:** 2022-08-11

**Authors:** Kunming Cheng, Qiang Guo, Zefeng Shen, Weiguang Yang, Yan Zhou, Zaijie Sun, Xiuhua Yao, Haiyang Wu

**Affiliations:** ^1^ Department of Intensive Care Unit, The Second Affiliated Hospital of Zhengzhou University, Zhengzhou, China; ^2^ Department of Orthopaedic Surgery, Baodi Clinical College of Tianjin Medical University, Tianjin, China; ^3^ Sun Yat-Sen Memorial Hospital, Graduate School of Sun Yat-sen University, Guangzhou, China; ^4^ Graduate School of Tianjin Medical University, Tianjin, China; ^5^ Clinical College of Neurology, Neurosurgery and Neurorehabilitation, Tianjin Medical University, Tianjin, China; ^6^ Department of Orthopaedic Surgery, Xiangyang Central Hospital, Affiliated Hospital of Hubei University of Arts and Science, Xiangyang, China; ^7^ Tianjin Key Laboratory of Cerebrovascular and Neurodegenerative Diseases, Tianjin Neurosurgical Institute, Tianjin Huanhu Hospital, Tianjin, China

**Keywords:** ferroptosis, citation, hotspot, bibliometric analysis, cancer

## Abstract

In recent years, ferroptosis has become a research hotspot in programmed cell death. Since the concept of ferroptosis was proposed, a growing number of articles have been published on this topic. Nevertheless, to our knowledge, these ferroptosis-related publications that have received a great deal of attention have not been quantitatively evaluated. In this study, we analyzed the top 100 most influential articles over the past decade through a bibliometric method to characterize the research status and trends in this field. Web of Science Core Collection was searched to identify relevant studies. After being manually screened, the top 100 most cited studies with original data were identified and analyzed. Bibliometric software including VOSviewer and R-Bibliometrix were used to perform visualization analysis. The citation frequency for the top 100 selected articles ranged from 135 to 3603 (326.6 citations on average). These articles originated from 25 countries/regions, with more than half originating from the United States and China. The most frequently nominated author was Stockwell BR from the Columbia University, and of the top 100 articles, 19 listed his name. Three core journals were *Nature*, *Cell* and *Proceedings of the National Academy of Sciences of the United States of America*. In addition to term of ferroptosis, these terms or phrases including cell death, cancer cell, GPX4, pathway, inhibitor, mechanism, iron, lipid peroxidation, resistance, erastin, sorafenib, P53, reactive oxygen species, necroptosis, apoptosis, glutathione peroxidase, ACSL4, autophagy, and SLC7A11 appeared more frequently in the top 100 articles. Overall, although much progress has been made, the research on ferroptosis is still at an early stage. The current attention in this field mainly focuses on potential regulatory mechanism and pathways including key ferroptosis-related genes/molecules, oxidant and antioxidant system, ferroptosis-inducing agents or nanomedicine for cancer therapy, as well as the role of ferroptosis in non-neoplastic disorders. Meanwhile, combination therapeutic strategies targeting ferroptosis in radiotherapy or immunotherapy also deserve further attention.

## Introduction

Cell death is generally classified into two main distinct categories: accidental cell death (ACD) and regulated cell death (RCD) (1). Unlike ACD, RCD follows multiple subroutines, which could be mediated through a series of molecular cascades and regulatory pathways. Although initial studies on RCD have focused on apoptosis, several other novel forms of non-apoptotic cell death such as ferroptosis, pyroptosis, necroptosis, NETosis, etc., have drawn extensive attention recently (2–4). Among them, ferroptosis, an iron-dependent cell death modality, is characterized by iron overload and lipid reactive oxygen species (ROS) accumulation (5, 6). Meanwhile, it displays unique morphological features such as shrinkage of the mitochondria, reduction or disappearance of mitochondrial ridges, and a ruptured outer mitochondrial membrane. Since the term “ferroptosis” was coined in 2012, a notable amount of works has been devoted to identifying the underlying regulation mechanisms and signaling pathways of ferroptosis (7). In brief, multiple ferroptosis-inducing factors are able to affect glutathione peroxidase directly or indirectly *via* different pathways, resulting in an imbalance between oxidant and antioxidant ability, and elevated lipid peroxidation in cells, ultimately leading to irreversible oxidative damage and cell death (8, 9). Lipid peroxidation products generated in this process can be pharmacologically inhibited by iron chelators (e.g., deferoxamine) or lipid peroxidation inhibitors (e.g., eugenol), and lipophilic antioxidants (e.g., ferrostatin-1, liproxstatin-1, vitamin E) (10–12). Besides, Glutathione, GSH peroxidase 4 (GPX4), nuclear factor erythroid 2-related factor 2 (Nrf2), heat shock protein beta-1 (HSPB1) exert negative regulatory roles in ferroptosis by limiting the production of ROS or altering cellular iron uptake (13–15).

**Figure 1:**
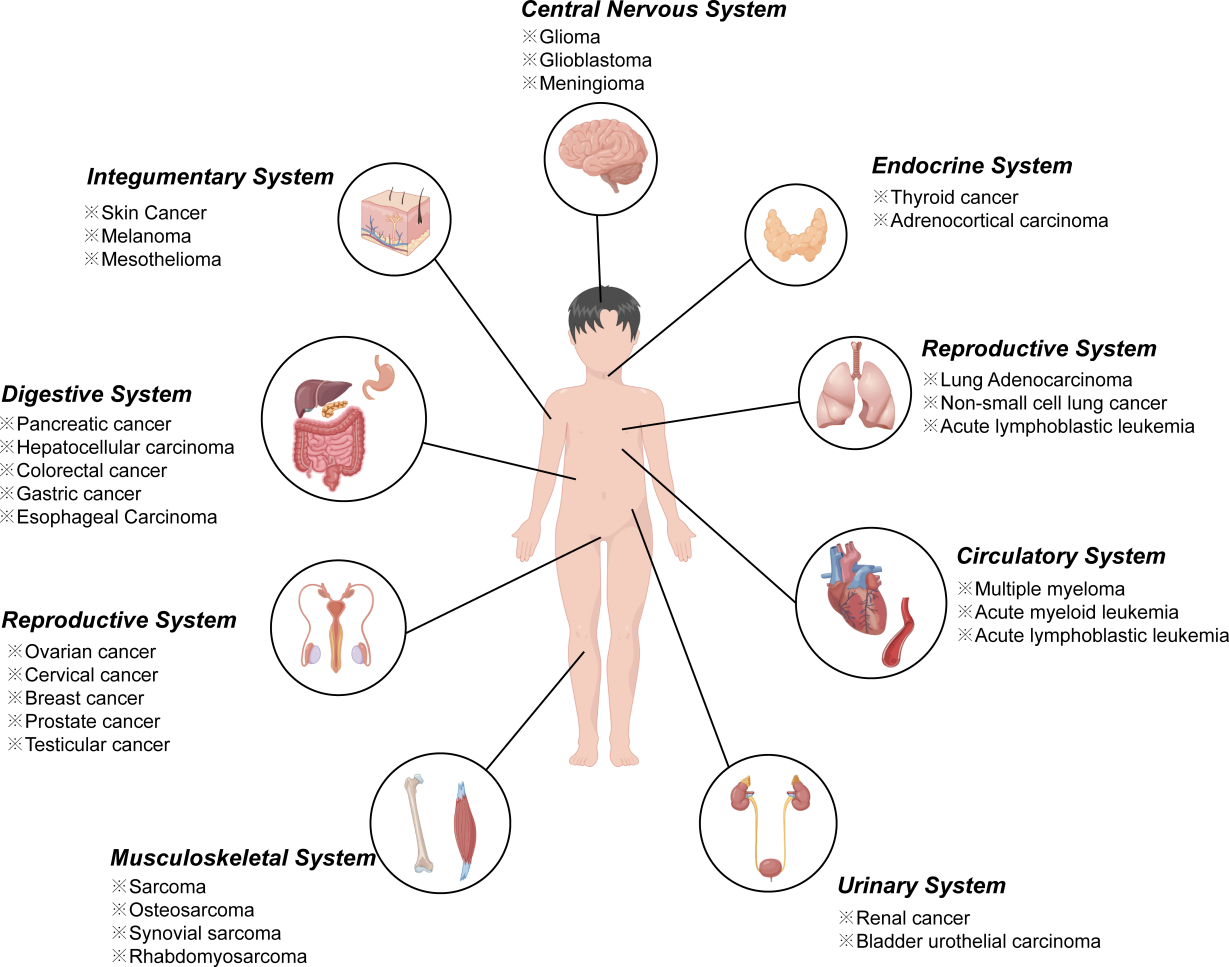
Ferroptosis in various cancers from different systems.

In recent year, an increasing number of studies have found that ferroptosis played an important regulatory role in the initiation and development of various diseases including almost all cancers (16–19) (**Figure 1**), ischemia reperfusion injury (20), neurological diseases (21–23), digestive disorders (24), hematological system diseases (25), as well as diseases of other systems. Take tumor diseases as examples, ferroptosis inducers such as erastin, sulfasalazine, and sorafenib, several conventional drugs or natural compounds have been extensively examined as novel anti-cancer therapeutics (26–28). In addition, further investigations revealed that erastin combining with traditional chemotherapeutic drugs including temozolomide, cisplatin, and doxorubicin offered remarkable synergistic therapeutic effect on their anti-tumor activity, compared with chemotherapy alone (29, 30).

In view of this, a plethora of work related to ferroptosis have been published and added our understanding to this field. Generally speaking, citation analysis is a widely accepted index for evaluating the impact of scientific articles (31, 32);. These studies with a higher number of citations are often considered as pioneering articles or hot research topics in an area. Thus, highly cited publications in a certain field could provide valuable evidence and information on research trends and scientific progress (33). In previous studies, many special fields have summarized the most-cited studies in their specialty with bibliometric method, such as hepatocellular carcinoma (34), gastric cancer (35), psychosomatic research (36), programmed cell death 1 (PD-1)/programmed cell death ligand 1 (PD-L1) inhibitors (37), metabolic disorders (38, 39), and so on. Nevertheless, no work has been published on the top 100 most cited studies regarding ferroptosis.

In this study, we analyzed the characteristics of these top 100 most influential papers in the field of ferroptosis over the past decade. In turn, the data could help researchers better understand the influential works in the evolution of the specialty, as well as provide meaningful insight to conduct further studies. To the best of our knowledge, this work is not the first to evaluate the publications in the field of ferroptosis with bibliometric methods (40), but is the first bibliometric analysis dedicated to ferroptosis related studies with high levels of influence.

## Materials and methods

### Search strategies

The top 100 most cited articles on ferroptosis were retrieved from Science Citation Index Expanded (SCIE) of Web of Science Core Collection (WoSCC, Clarivate Analytics, Philadelphia, PA, USA) on the same day. Data were collected based on the titles (TI), abstracts (AB) and author keywords (AK) with the following strategy: TI=(ferroptosis OR ferroptotic) OR AK=(ferroptosis OR ferroptotic) OR AB=(ferroptosis OR ferroptotic). The period of the publication for selection was from January 2012 to May 2022, with no limitations on languages.

### Data extraction

Firstly, records were placed in descending order based on their frequency of citations in WoSCC. Two researchers (CKM and GQ) independently screened the title, abstract and document type, if necessary, reading the full article for a more detailed assessment, to confirm if it should be included. The inclusion criterion was ferroptosis related studies with original data. Review articles or meta-analysis without original data were excluded. To our knowledge, some reviews articles have been mis-annotated as articles in WoSCC. Discrepancies were resolved *via* discussion until the two researchers reached an agreement under the verification of senior experts. Then, these top 100 articles were downloaded and exported in plain text format for further analysis. A similar procedure was employed to identify the top 50 highly-cited reviews. Journal impact factors were obtained from 2021 Journal Citation Reports. The citation density of each article was defined as the average citations since published, that is the ratio of total citations and literature age (41).

### Statistical analysis

Microsoft Excel 2019 and R software (v 4.1.0) software was used for descriptive statistical analysis and generating diagrams. VOSviewer 1.6.16 (Leiden University, the Netherlands) software were used to perform country/institutional co-authorship analysis, author/journal co-citation analysis, terms co-occurrence analysis. VOSviewer, developed by van Eck and Waltman, is a literature knowledge visualization software for constructing bibliometric networks (42). In the network visualization maps, different nodes represent different elements such as countries, institutions, authors or terms. The links between nodes represent relationships such as co-authorship, co-citation or co-occurrence, and weighted by total link strength (TLS) (43). Co-authorship analysis measures collaboration links between countries or institutions. The relatedness of nodes is determined based on the number of coauthored documents. While co-citation analysis measures the relationship among nodes based on the times they are cited by the same document. As for co-occurrence analysis, the relatedness of nodes is determined according to the number of documents in which they occur together (42). The size of the nodes reflects the number of outputs, citations or occurrences and the color indicates different clusters or average appearing year (AAY) of these elements. The “bibliometrix” package of R software was used for mapping historical direct citation network and cloud map of author keywords (44).

## Results

**Figure 2:**
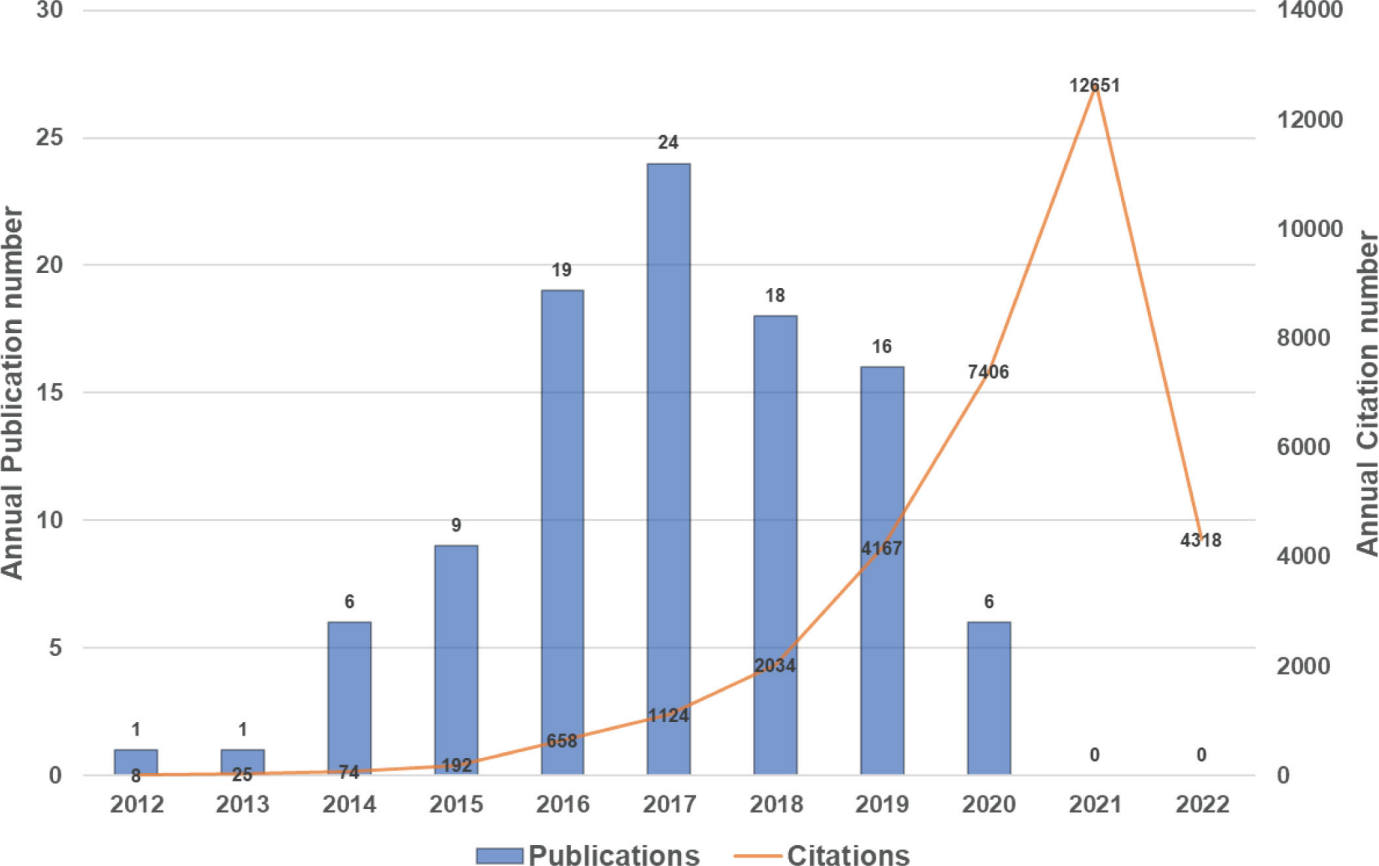
Number of top-cited publications from 2012 to 2020.

The top 100 highly cited articles are listed in descending order based on their total citation number in **Table 1**. The citation frequency received by these 100 studies ranged from 135 to 3603 (mean: 326.6). About half of the articles (n=54) received more than 200 citations, and only 3 articles were cited more than 1000 times. The citation density of each article was ranged from 18.56 to 327.55 (mean: 53.1, **Supplementary Figure 1**). As is apparent, the pioneering study titled “Ferroptosis: An Iron-Dependent Form of Nonapoptotic Cell Death”, by Dixon SJ et al., published in *Cell* in 2012, has received the most citations either total citation or adjusted citation count over the past 11 years. As shown in **Figure 2**, the top100 articles were published between 2012 and 2020. The year that yielded the greatest number of high-impact articles was in 2017 (n=24), followed by the year in 2016 (n=19), and 2018 (n=18). Additionally, the top 50 highly cited reviews on ferroptosis were summarized in **Supplementary Table1**.

**Table 1:** Top 100 highly cited articles on ferroptosis ranked according to their total citations counts.

Ranking	Title	Total citations	AC per year	Journal	First Author	Publishedyear
1	Ferroptosis: An Iron-Dependent Form of Nonapoptotic Cell Death	3603	327.55	CELL	Dixon, Scott J	2012
2	Regulation of Ferroptotic Cancer Cell Death by GPX4	1845	205	CELL	Yang, Wan Seok	2014
3	Inactivation of the ferroptosis regulator Gpx4 triggers acute renal failure in mice	1068	118.67	NATURE CELL BIOLOGY	Angeli, Jose Pedro Friedmann	2014
4	Ferroptosis as a p53-mediated activity during tumour suppression	954	119.25	NATURE	Jiang, Le	2015
5	ACSL4 dictates ferroptosis sensitivity by shaping cellular lipid composition	807	134.5	NATURE CHEMICAL BIOLOGY	Doll, Sebastian	2017
6	Oxidized arachidonic and adrenic PEs navigate cells to ferroptosis	681	113.5	NATURE CHEMICAL BIOLOGY	Kagan, Valerian E	2017
7	Pharmacological inhibition of cystine-glutamate exchange induces endoplasmic reticulum stress and ferroptosis	669	74.33	ELIFE	Dixon, Scott J	2014
8	Glutaminolysis and Transferrin Regulate Ferroptosis	658	82.25	MOLECULAR CELL	Gao, Minghui	2015
9	Activation of the p62-Keap1-NRF2 pathway protects against ferroptosis in hepatocellular carcinoma cells	600	85.71	HEPATOLOGY	Sun, Xiaofang	2016
10	Autophagy promotes ferroptosis by degradation of ferritin	574	82	AUTOPHAGY	Hou, Wen	2016
11	The CoQ oxidoreductase FSP1 acts parallel to GPX4 to inhibit ferroptosis	564	141	NATURE	Bersuker, Kirill	2019
12	FSP1 is a glutathione-independent ferroptosis suppressor	562	140.5	NATURE	Doll, Sebastian	2019
13	Peroxidation of polyunsaturated fatty acids by lipoxygenases drives ferroptosis	551	78.71	PROCEEDINGS OF THE NATIONAL ACADEMY OF SCIENCES OF THE UNITED STATES OF AMERICA	Yang, Wan Seok	2016
14	CD8(+) T cells regulate tumour ferroptosis during cancer immunotherapy	548	137	NATURE	Wang, Weimin	2019
15	Dependency of a therapy-resistant state of cancer cells on a lipid peroxidase pathway	544	90.67	NATURE	Viswanathan, Vasanthi S	2017
16	Ferroptosis is an autophagic cell death process	481	68.71	CELL RESEARCH	Gao, Minghui	2016
17	Synchronized renal tubular cell death involves ferroptosis	476	52.89	PROCEEDINGS OF THE NATIONAL ACADEMY OF SCIENCES OF THE UNITED STATES OF AMERICA	Linkermann, Andreas	2014
18	Drug-tolerant persister cancer cells are vulnerable to GPX4 inhibition	453	75.5	NATURE	Hangauer, Matthew J	2017
19	Ferroptosis as a target for protection against cardiomyopathy	441	110.25	PROCEEDINGS OF THE NATIONAL ACADEMY OF SCIENCES OF THE UNITED STATES OF AMERICA	Fang, Xuexian	2019
20	Ferrostatins Inhibit Oxidative Lipid Damage and Cell Death in Diverse Disease Models	424	47.11	JOURNAL OF THE AMERICAN CHEMICAL SOCIETY	Skouta, Rachid	2014
21	Selenium Utilization by GPX4 Is Required to Prevent Hydroperoxide-Induced Ferroptosis	387	77.4	CELL	Ingold, Irina	2018
22	Role of Mitochondria in Ferroptosis	355	88.75	MOLECULAR CELL	Gao, Minghui	2019
23	Global survey of cell death mechanisms reveals metabolic regulation of ferroptosis	318	45.43	NATURE CHEMICAL BIOLOGY	Shimada, Kenichi	2016
24	Ultrasmall nanoparticles induce ferroptosis in nutrient-deprived cancer cells and suppress tumour growth	295	42.14	NATURE NANOTECHNOLOGY	Kim, Sung Eun	2016
25	PEBP1 Wardens Ferroptosis by Enabling Lipoxygenase Generation of Lipid Death Signals	291	48.5	CELL	Wenzel, Sally E	2017
26	Human Haploid Cell Genetics Reveals Roles for Lipid Metabolism Genes in Nonapoptotic Cell Death	289	36.13	ACS CHEMICAL BIOLOGY	Dixon, Scott J	2015
27	Ablation of ferroptosis regulator glutathione peroxidase 4 in forebrain neurons promotes cognitive impairment and neurodegeneration	284	47.33	REDOX BIOLOGY	Hambright, William Sealy	2017
28	The Tumor Suppressor p53 Limits Ferroptosis by Blocking DPP4 Activity	281	46.83	CELL REPORTS	Xie, Yangchun	2017
29	Identification of ACSL4 as a biomarker and contributor of ferroptosis	269	38.43	BIOCHEMICAL AND BIOPHYSICAL RESEARCH COMMUNICATIONS	Yuan, Hua	2016
30	On the Mechanism of Cytoprotection by Ferrostatin-1 and Liproxstatin-1 and the Role of Lipid Peroxidation in Ferroptotic Cell Death	267	44.5	ACS CENTRAL SCIENCE	Zilka, Omkar	2017
31	BAP1 links metabolic regulation of ferroptosis to tumour suppression	266	53.2	NATURE CELL BIOLOGY	Zhang, Yilei	2018
32	Inhibition of neuronal ferroptosis protects hemorrhagic brain	260	43.33	JCI INSIGHT	Li, Qian	2017
33	Ferroptosis, a newly characterized form of cell death in Parkinson’s disease that is regulated by PKC	256	36.57	NEUROBIOLOGY OF DISEASE	Do Van, Bruce	2016
34	T cell lipid peroxidation induces ferroptosis and prevents immunity to infection	255	31.88	JOURNAL OF EXPERIMENTAL MEDICINE	Matsushita, Mai	2015
35	Multi-stage Differentiation Defines Melanoma Subtypes with Differential Vulnerability to Drug-Induced Iron-Dependent Oxidative Stress	253	50.6	CANCER CELL	Tsoi, Jennifer	2018
36	Cysteine depletion induces pancreatic tumor ferroptosis in mice	238	79.33	SCIENCE	Badgley, Michael A	2020
37	Selenium Drives a Transcriptional Adaptive Program to Block Ferroptosis and Treat Stroke	236	59	CELL	Alim, Ishraq	2019
38	NFS1 undergoes positive selection in lung tumours and protects cells from ferroptosis	234	39	NATURE	Alvarez, Samantha W	2017
39	Metallothionein-1G Facilitates Sorafenib Resistance Through Inhibition of Ferroptosis	229	32.71	HEPATOLOGY	Sun, Xiaofang	2016
40	Salinomycin kills cancer stem cells by sequestering iron in lysosomes	226	37.67	NATURE CHEMISTRY	Trang Thi Mai	2017
41	Neuronal Death After Hemorrhagic Stroke *In Vitro* and *In Vivo* Shares Features of Ferroptosis and Necroptosis	224	37.33	STROKE	Zille, Marietta	2017
42	Activation of SAT1 engages polyamine metabolism with p53-mediated ferroptotic responses	224	32	PROCEEDINGS OF THE NATIONAL ACADEMY OF SCIENCES OF THE UNITED STATES OF AMERICA	Ou, Yang	2016
43	Heme oxygenase-1 accelerates erastin-induced ferroptotic cell death	223	27.88	ONCOTARGET	Kwon, Min-Young	2015
44	Fenton-Reaction-Acceleratable Magnetic Nanoparticles for Ferroptosis Therapy of Orthotopic Brain Tumors	222	44.4	ACS NANO	Shen, Zheyu	2018
45	FINO2 initiates ferroptosis through GPX4 inactivation and iron oxidation	222	44.4	NATURE CHEMICAL BIOLOGY	Gaschler, Michael M	2018
46	Iron-dependent cell death of hepatocellular carcinoma cells exposed to sorafenib	221	22.1	INTERNATIONAL JOURNAL OF CANCER	Louandre, Christophe	2013
47	Tau-mediated iron export prevents ferroptotic damage after ischemic stroke	217	36.17	MOLECULAR PSYCHIATRY	Tuo, Q-z	2017
48	HSPB1 as a novel regulator of ferroptotic cancer cell death	217	27.13	ONCOGENE	Sun, X	2015
49	Switching Apoptosis to Ferroptosis: Metal-Organic Network for High-Efficiency Anticancer Therapy	215	35.83	NANO LETTERS	Zheng, Di-Wei	2017
50	Intercellular interaction dictates cancer cell ferroptosis via NF2-YAP signalling	214	53.5	NATURE	Wu, Jiao	2019
51	Characterization of Ferroptosis in Murine Models of Hemochromatosis	211	35.17	HEPATOLOGY	Wang, Hao	2017
52	Nrf2 inhibition reverses the resistance of cisplatin-resistant head and neck cancer cells to artesunate-induced ferroptosis	210	35	REDOX BIOLOGY	Roh, Jong-Lyel	2017
53	Radiotherapy and Immunotherapy Promote Tumoral Lipid Oxidation and Ferroptosis via Synergistic Repression of SLC7A11	209	52.25	CANCER DISCOVERY	Lang, Xueting	2019
54	Ferrous-Supply-Regeneration Nanoengineering for Cancer-Cell-Specific Ferroptosis in Combination with Imaging-Guided Photodynamic Therapy	200	40	ACS NANO	Liu, Tao	2018
55	Ferroptosis is induced following siramesine and lapatinib treatment of breast cancer cells	199	28.43	CELL DEATH & DISEASE	Ma, S	2016
56	Ablation of the Ferroptosis Inhibitor Glutathione Peroxidase 4 in Neurons Results in Rapid Motor Neuron Degeneration and Paralysis	199	24.88	JOURNAL OF BIOLOGICAL CHEMISTRY	Chen, Liuji	2015
57	Nrf2-Keap1 pathway promotes cell proliferation and diminishes ferroptosis	198	33	ONCOGENESIS	Fan, Z	2017
58	AMPK-Mediated BECN1 Phosphorylation Promotes Ferroptosis by Directly Blocking System X-c(-) Activity	195	39	CURRENT BIOLOGY	Song, Xinxin	2018
59	p53 Suppresses Metabolic Stress-Induced Ferroptosis in Cancer Cells	193	38.6	CELL REPORTS	Tarangelo, Amy	2018
60	A GPX4-dependent cancer cell state underlies the clear-cell morphology and confers sensitivity to ferroptosis	192	48	NATURE COMMUNICATIONS	Zou, Yilong	2019
61	Resolving the Role of Lipoxygenases in the Initiation and Execution of Ferroptosis	188	37.6	ACS CENTRAL SCIENCE	Shah, Ron	2018
62	Ischemia-induced ACSL4 activation contributes to ferroptosis-mediated tissue injury in intestinal ischemia/reperfusion	186	46.5	CELL DEATH AND DIFFERENTIATION	Li, Yang	2019
63	Long noncoding RNA LINC00336 inhibits ferroptosis in lung cancer by functioning as a competing endogenous RNA	186	46.5	CELL DEATH AND DIFFERENTIATION	Wang, Min	2019
64	Loss of cysteinyl-tRNA synthetase (CARS) induces the transsulfuration pathway and inhibits ferroptosis induced by cystine deprivation	186	26.57	CELL DEATH AND DIFFERENTIATION	Hayano, M	2016
65	Nanocatalytic Tumor Therapy by Single-Atom Catalysts	184	46	ACS NANO	Huo, Minfeng	2019
66	The role of ferroptosis in ionizing radiation-induced cell death and tumor suppression	183	61	CELL RESEARCH	Lei, Guang	2020
67	Artemisinin derivatives induce iron-dependent cell death (ferroptosis) in tumor cells	181	22.63	PHYTOMEDICINE	Ooko, Edna	2015
68	Identification and Successful Negotiation of a Metabolic Checkpoint in Direct Neuronal Reprogramming	180	25.71	CELL STEM CELL	Gascon, Sergio	2016
69	Nano-targeted induction of dual ferroptotic mechanisms eradicates high-risk neuroblastoma	179	35.8	JOURNAL OF CLINICAL INVESTIGATION	Hassannia, Behrouz	2018
70	Ferroptosis, but Not Necroptosis, Is Important in Nephrotoxic Folic Acid-Induced AKI	178	29.67	JOURNAL OF THE AMERICAN SOCIETY OF NEPHROLOGY	Martin-Sanchez, Diego	2017
71	Exogenous Monounsaturated Fatty Acids Promote a Ferroptosis-Resistant Cell State	177	44.25	CELL CHEMICAL BIOLOGY	Magtanong, Leslie	2019
72	ALOX12 is required for p53-mediated tumour suppression through a distinct ferroptosis pathway	175	43.75	NATURE CELL BIOLOGY	Chu, Bo	2019
73	Mitochondrial complex I inhibition triggers a mitophagy-dependent ROS increase leading to necroptosis and ferroptosis in melanoma cells	171	28.5	CELL DEATH & DISEASE	Basit, Farhan	2017
74	Ferroptosis: A Novel Anti-tumor Action for Cisplatin	170	34	CANCER RESEARCH AND TREATMENT	Guo, Jipeng	2018
75	CISD1 inhibits ferroptosis by protection against mitochondrial lipid peroxidation	170	24.29	BIOCHEMICAL AND BIOPHYSICAL RESEARCH COMMUNICATIONS	Yuan, Hua	2016
76	CAF secreted miR-522 suppresses ferroptosis and promotes acquired chemo-resistance in gastric cancer	169	56.33	MOLECULAR CANCER	Zhang, Haiyang	2020
77	Sorafenib Induces Ferroptosis in Human Cancer Cell Lines Originating from Different Solid Tumors	167	18.56	ANTICANCER RESEARCH	Lachaier, Emma	2014
78	An African-specific polymorphism in the TP53 gene impairs p53 tumor suppressor function in a mouse model	165	23.57	GENES & DEVELOPMENT	Jennis, Matthew	2016
79	Arginine-Rich Manganese Silicate Nanobubbles as a Ferroptosis-Inducing Agent for Tumor-Targeted Theranostics	164	32.8	ACS NANO	Wang, Shuaifei	2018
80	Acetylation Is Crucial for p53-Mediated Ferroptosis and Tumor Suppression	164	23.43	CELL REPORTS	Wang, Shang-Jui	2016
81	Imidazole Ketone Erastin Induces Ferroptosis and Slows Tumor Growth in a Mouse Lymphoma Model	159	39.75	CELL CHEMICAL BIOLOGY	Zhang, Yan	2019
82	Energy-stress-mediated AMPK activation inhibits ferroptosis	158	52.67	NATURE CELL BIOLOGY	Lee, Hyemin	2020
83	A G3BP1-Interacting lncRNA Promotes Ferroptosis and Apoptosis in Cancer via Nuclear Sequestration of p53	158	31.6	CANCER RESEARCH	Mao, Chao	2018
84	HSPA5 Regulates Ferroptotic Cell Death in Cancer Cells	158	26.33	CANCER RESEARCH	Zhu, Shan	2017
85	The retinoblastoma (Rb) protein regulates ferroptosis induced by sorafenib in human hepatocellular carcinoma cells	158	19.75	CANCER LETTERS	Louandre, Christophe	2015
86	Lymph protects metastasizing melanoma cells from ferroptosis	157	52.33	NATURE	Ubellacker, Jessalyn M	2020
87	An essential role for functional lysosomes in ferroptosis of cancer cells	154	22	BIOCHEMICAL JOURNAL	Torii, Seiji	2016
88	Glutathione depletion induces ferroptosis, autophagy, and premature cell senescence in retinal pigment epithelial cells	152	30.4	CELL DEATH & DISEASE	Sun, Yun	2018
89	miR-137 regulates ferroptosis by targeting glutamine transporter SLC1A5 in melanoma	150	30	CELL DEATH AND DIFFERENTIATION	Luo, Meiying	2018
90	Heme oxygenase-1 mediates BAY 11-7085 induced ferroptosis	150	30	CANCER LETTERS	Chang, Ling-Chu	2018
91	Necroptosis and ferroptosis are alternative cell death pathways that operate in acute kidney failure	144	24	CELLULAR AND MOLECULAR LIFE SCIENCES	Mueller, Tammo	2017
92	Heme oxygenase-1 mitigates ferroptosis in renal proximal tubule cells	143	28.6	AMERICAN JOURNAL OF PHYSIOLOGY-RENAL PHYSIOLOGY	Adedoyin, Oreoluwa	2018
93	Iron addiction: a novel therapeutic target in ovarian cancer	142	23.67	ONCOGENE	Basuli, D	2017
94	Quantitative real-time imaging of glutathione	140	23.33	NATURE COMMUNICATIONS	Jiang, Xiqian	2017
95	Chaperone-mediated autophagy is involved in the execution of ferroptosis	138	34.5	PROCEEDINGS OF THE NATIONAL ACADEMY OF SCIENCES OF THE UNITED STATES OF AMERICA	Wu, Zheming	2019
96	An Endoperoxide Reactivity-Based FRET Probe for Ratiometric Fluorescence Imaging of Labile Iron Pools in Living Cells	137	19.57	JOURNAL OF THE AMERICAN CHEMICAL SOCIETY	Aron, Allegra T	2016
97	A Novel Ferroptosis-related Gene Signature for Overall Survival Prediction in Patients with Hepatocellular Carcinoma	136	45.33	INTERNATIONAL JOURNAL OF BIOLOGICAL SCIENCES	Liang, Jie-ying	2020
98	Nrf2 inhibition reverses resistance to GPX4 inhibitor-induced ferroptosis in head and neck cancer	136	27.2	FREE RADICAL BIOLOGY AND MEDICINE	Shin, Daiha	2018
99	Lipoxygenase-mediated generation of lipid peroxides enhances ferroptosis induced by erastin and RSL3	135	22.5	CANCER SCIENCE	Shintoku, Ryosuke	2017
100	Dihydroartemisinin (DHA) induces ferroptosis and causes cell cycle arrest in head and neck carcinoma cells	135	19.29	CANCER LETTERS	Lin, Renyu	2016

AC, average citation.

**Figure 3:**
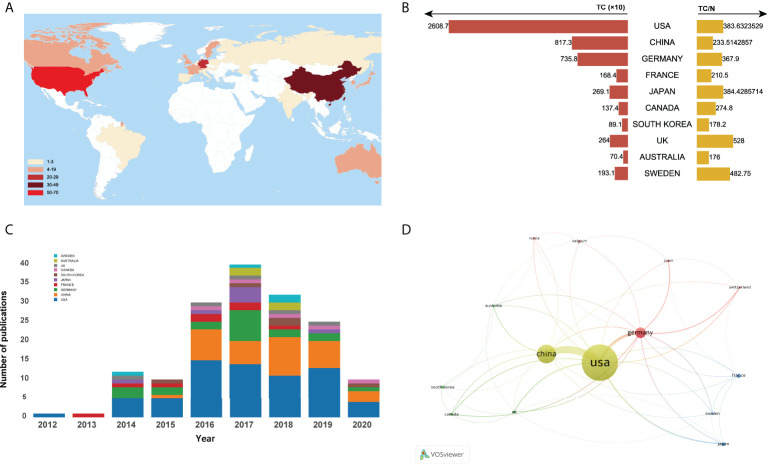
**(A)** Number of top-cited publications by countries/regions. **(B)**Total citations and average citations per document of top 10 most prolific countries/regions. **(C)** The number of annual publications of top 10 countries/regions on ferroptosis research from 2000 to 2020. **(D)** Network visualization map of country co-authorship analysis.

A total of 25 countries/regions worldwide contributed to the top 100 most cited articles, and 12 countries/regions had more than 3 articles, as shown in **Figure 3A**. Of them, the United States dominated the area with 68 articles and 26087 citations (shown in **Figure 3B**). China ranked second with 35 articles and 8173 citations, followed by Germany (20 articles and 7358 citations), France (8 articles and 1684 citations), and Japan (7 articles and 2691 citations). **Figure 3C** depicted the annual number of publications among the top 10 countries with the most outputs from 2012 to 2020. In addition, the network visualization map of co-authorship analysis among countries/regions, which could illustrate the collaboration network of countries/regions was conducted by VOSviewer in **Figure 3D**.

**Figure 4:**
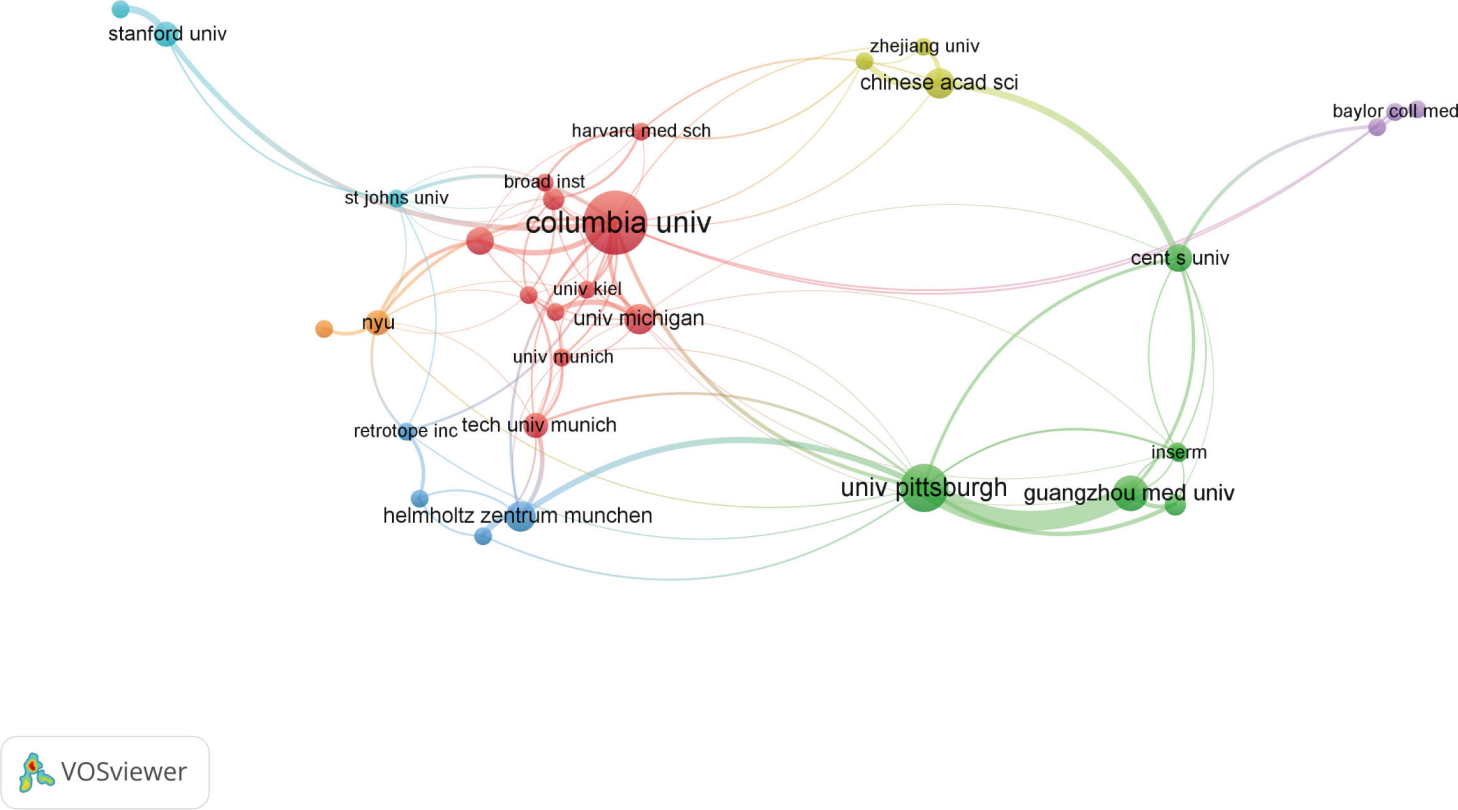
Network visualization map of institution co-authorship analysis.

As for institutions, more than 200 institutions contributed to the top 100 research. Of them, Columbia University produced the most top-cited articles (n=25), followed by University of Pittsburgh (n=15). As for institutional co-authorship analysis in **Figure 4**, only institutions with more than 3 documents were included. Of the 33 institutions met the threshold, Columbia University, University of Pittsburgh, and Guangzhou Medical University were the top 3 institutions with the largest TLS.

**Figure 5:**
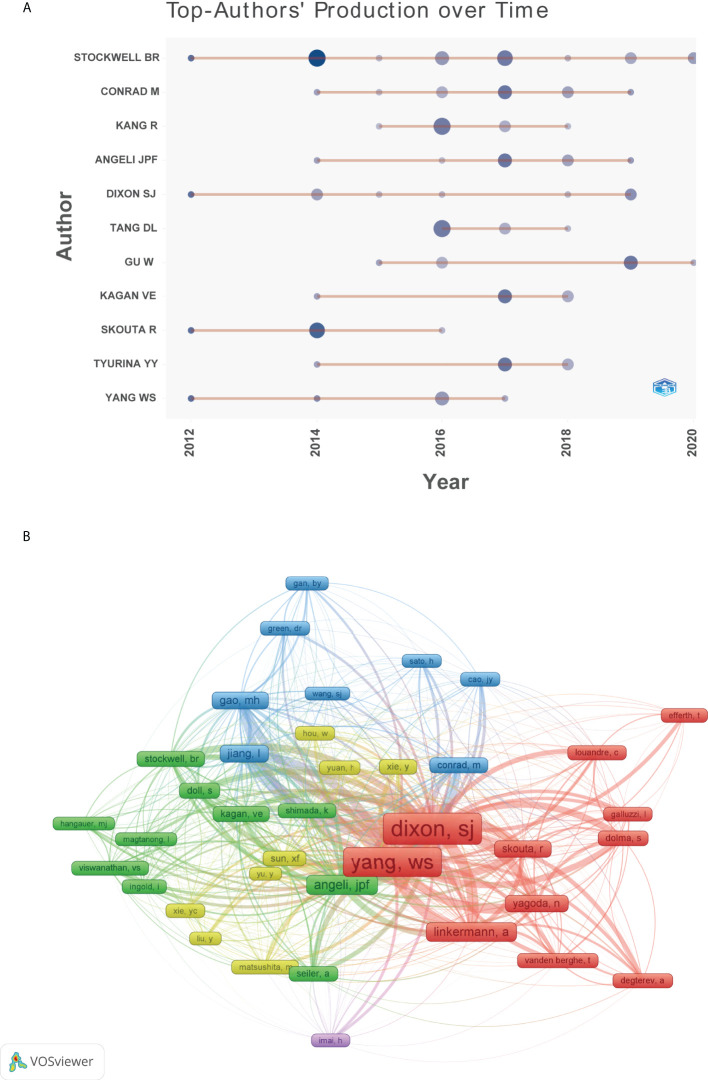
**(A)** Top 10 authors’ production over time. **(B)** Network visualization map of author co-citation analysis.

The top 10 authors involved in the top 100 articles were listed in **Table 2**. The list was led by Stockwell BR from the Columbia University, who participated 19 of the top 100 articles. Meanwhile, four other authors in the list also came from this university. **Figure 5A** depicted the annual outputs of these top 10 authors between 2012 and 2020. As for author co-citation analysis in **Figure 5B**, Dixon SJ, Yang WS, and Angeli JPF were the top three authors with the greatest TLS, and occupied the central position in the network map.

**Table 2:** Top 10 authors with the most publications.

Ranking	Author	Publications, n	TC	TC/N	Institution	Country
1	Stockwell BR	19	12049	634.16	Columbia University	USA
2	Conrad M	10	4681	468.1	Helmholtz Zentrum München	Germany
3	Kang R	9	2693	299.22	University of Pittsburgh	USA
4	Angeli JPF	8	4131	516.38	Helmholtz Zentrum München	Germany
5	Dixon SJ	8	6237	779.63	Columbia University	USA
6	Tang DL	8	2476	309.5	University of Pittsburgh/Guangzhou Medical University	USA/China
7	Gu W	7	2744	392	Columbia University	USA
8	Kagan VE	6	3248	541.33	University of Pittsburgh	USA
9	Skouta R	6	7335	1222.5	Columbia University	USA
10*	Tyurina YY	6	3248	541.33	University of Pittsburgh	USA
10*	Yang WS	6	7047	1174.5	Columbia University	USA

Ranking, according to the number of total publications; TC, total citation; *tied for tenth.

**Figure 6:**
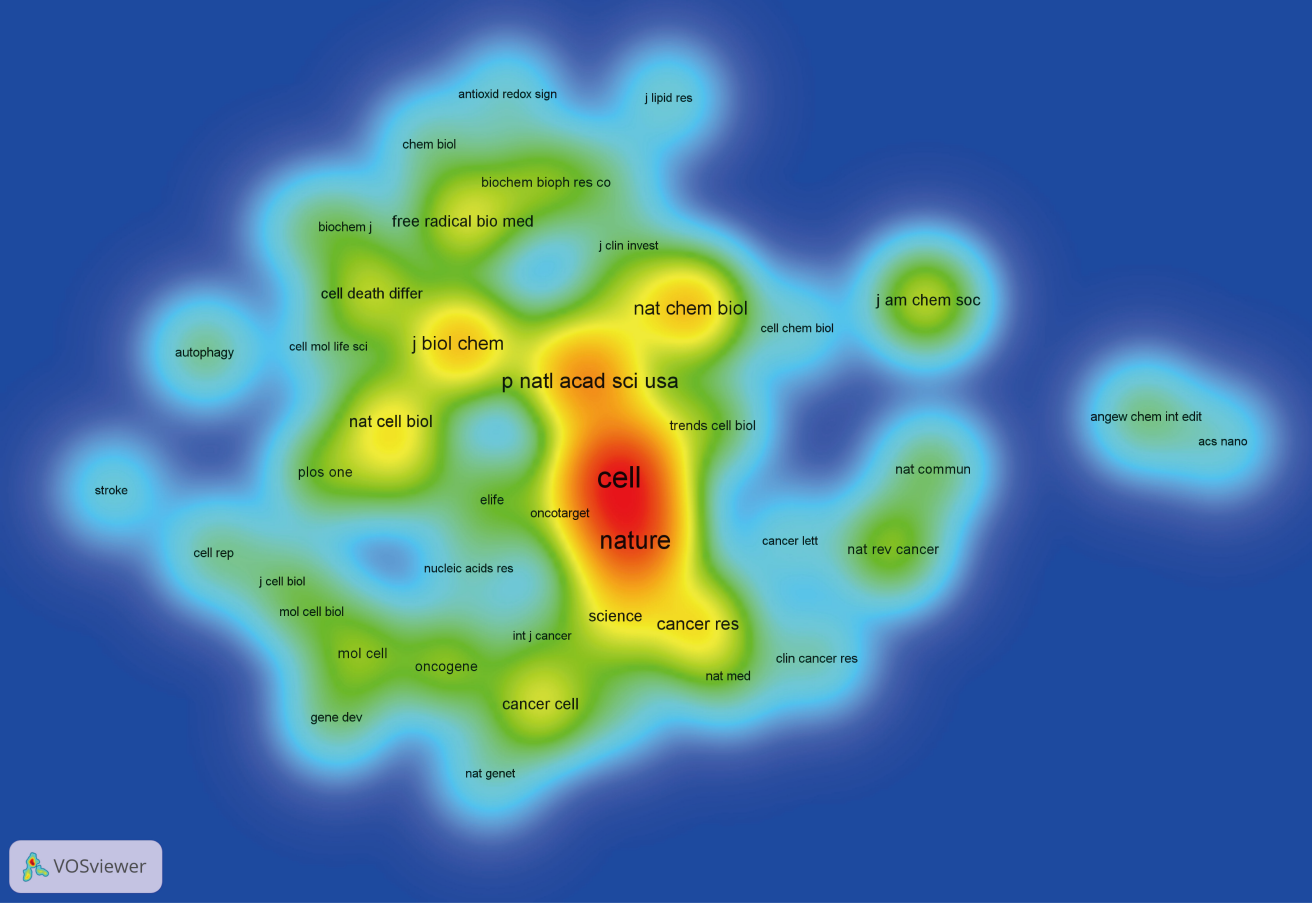
Density visualization map of journal co-citation analysis.

The top 100 articles were published in 54 different journals. Journals with more than 2 publications were listed in **Table 3** with their citations and impact factors. *Nature* published the most (n=9), followed by *Cell* (n=5) and *Proceedings of the National Academy of Sciences of the United States of America* (n=5). Journal co-citation analysis was conducted in **Figure 6**. According to the density visualization map, *Nature*, *Cell*, and *Proceedings of the National Academy of Sciences of the United States of America* were also the top 3 most co-citation journals in this field.

**Table 3:** Top 10 journals with the most publications.

Ranking	Sources Title	Publications, n	TC	TC/N	IF 2021
1	*NATURE*	9	4230	470	69.504
2	*CELL*	5	6362	1272.4	66.850
3	*PROCEEDINGS OF THE NATIONAL ACADEMY OF SCIENCES OF THE UNITED STATES OF AMERICA*	5	1830	366	12.779
4	*ACS NANO*	4	770	192.5	18.027
5	*CELL DEATH AND DIFFERENTIATION*	4	708	177	12.077
6	*NATURE CELL BIOLOGY*	4	1667	416.75	28.213
7	*NATURE CHEMICAL BIOLOGY*	4	2028	507	16.290
8	*CELL DEATH DISEASE*	3	522	174	9.705
9	*CELL REPORTS*	3	638	212.67	9.995
10*	*HEPATOLOGY*	3	1040	346.67	17.298
10*	*CANCER LETTERS*	3	443	147.67	9.756

Ranking: according to the number of total publications; TC, total citation; *tied for tenth.

**Figure 7:**
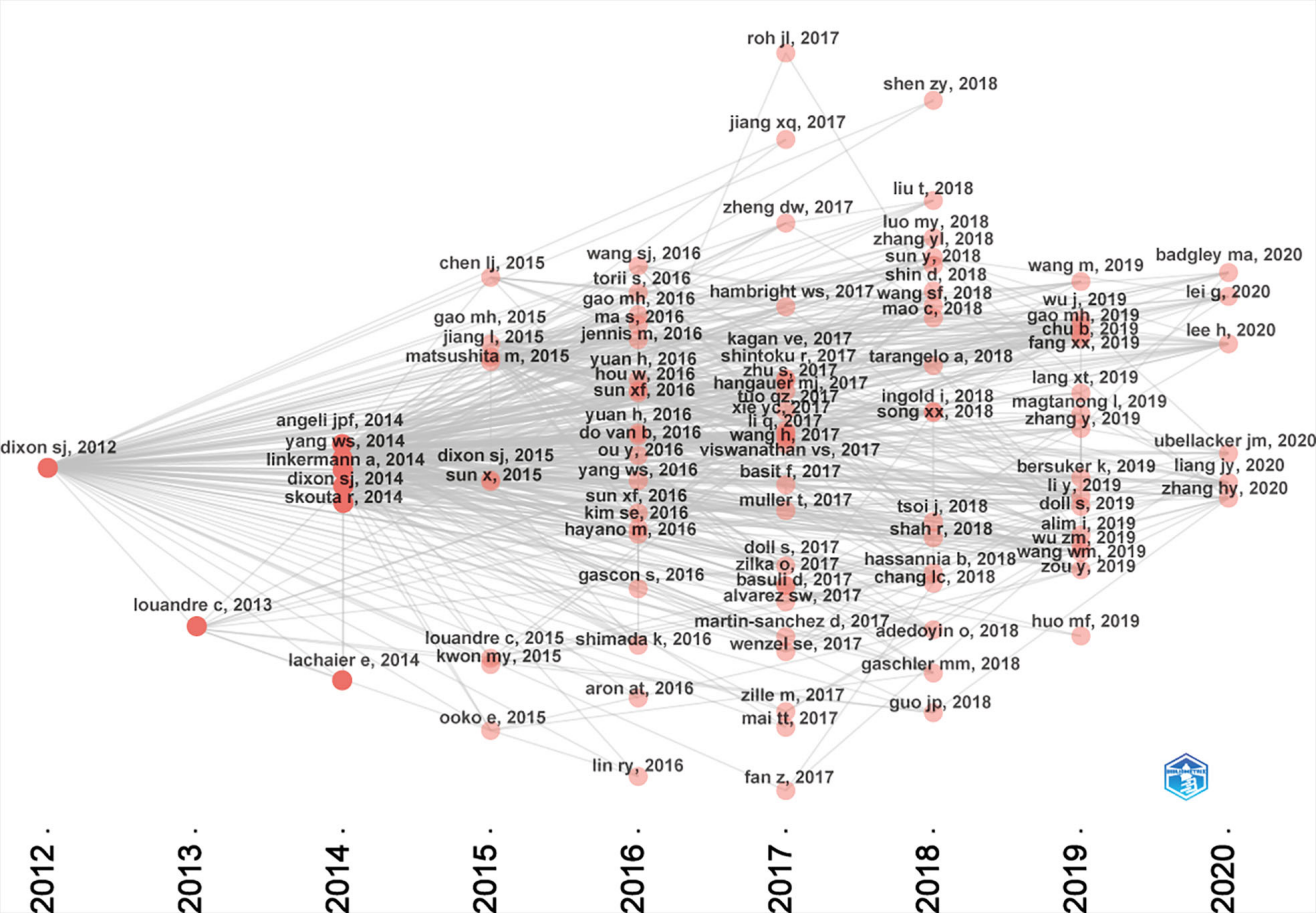
Historical direct citation network map of the top 100 articles.

**Figure 8:**
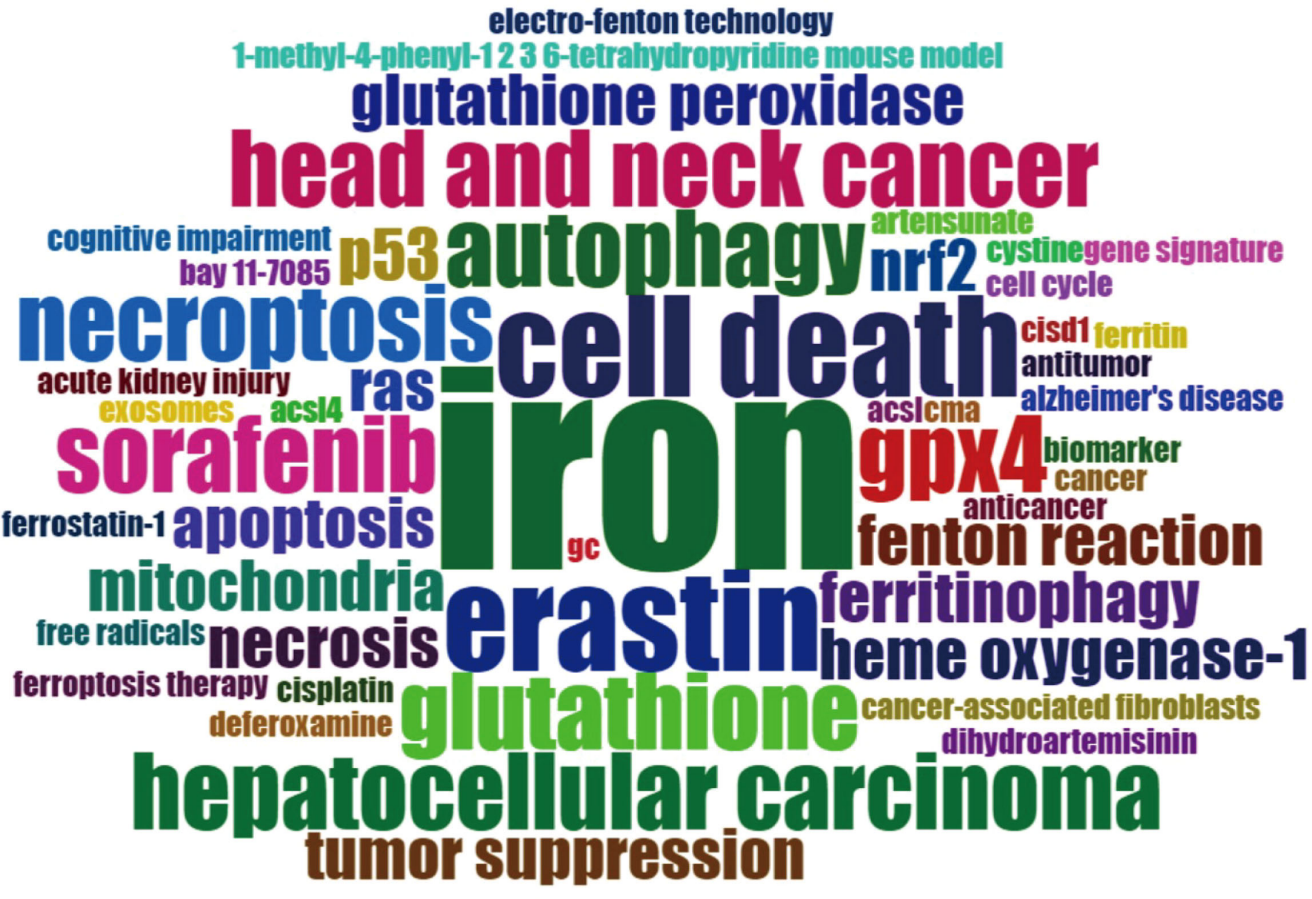
Cloud map of author keywords. Note: the keyword of “ferroptosis” was not included in this map.

**Figure 9:**
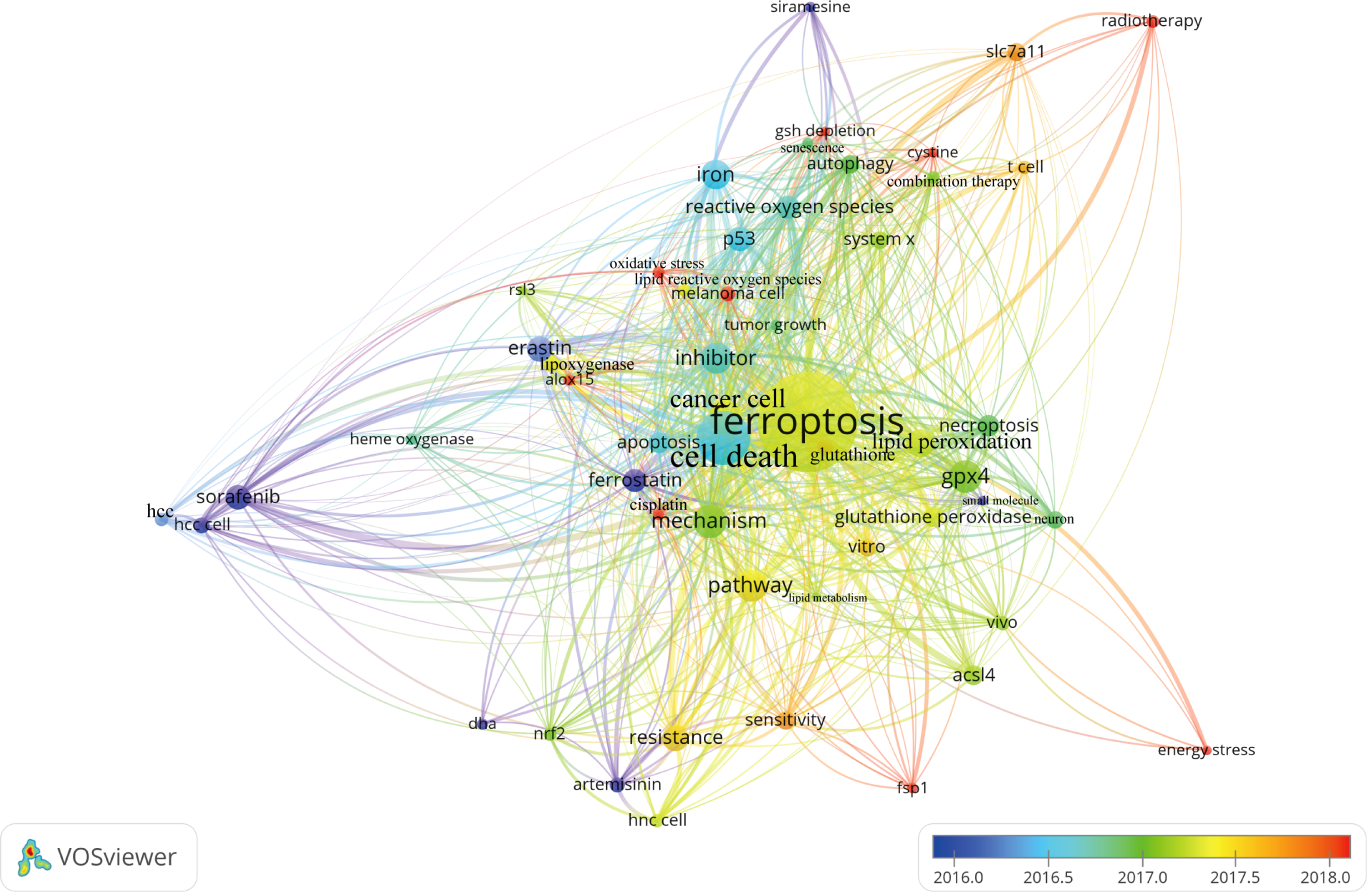
Overlay visualization map of terms generated with words from titles and abstracts by VOSviewer. Each node represents a term or phrase and the node size is proportional to occurrences. Distances between nodes indicates relatedness of words in terms of co-occurrence links. Different terms were given different colors based on their AAY.

**Figure 7** presented the historical direct citation network map of these top 100 articles. It can be seen that there are extensive connections among these studies. In addition, we also analyzed the terms or phrases appeared in the author keywords, titles and abstracts. A cloud map of author keywords is shown in **Figure 8**, apart from the keywords of ferroptosis (n=27), iron (n=8), reactive oxygen species (n=5), cell death (n=4), erastin (n=4), GPX4 (n=3), autophagy (n=3), hepatocellular carcinoma (n=3), head and neck cancer (n=3), necroptosis (n=3), and sorafenib (n=3) were these keywords with more than 3 times of frequency. As for network visualization map using terms from the titles and abstracts of the 100 most-cited articles, terms or phrases with a minimum of 7 occurrences were included (**Figure 9**). The 30 most frequent occurrences terms or phrases were listed in **Table 4**. In addition, all the included terms or phrases were scored according to the average publication year of the publications.

**Table 4:** Top 30 most frequent occurrences terms in titles and abstracts.

Ranking	Keywords	Occurrences	AAY	Ranking	Keywords	Occurrences	AAY
1	ferroptosis	483	2017.28	16	necroptosis	28	2016.93
2	cell death	116	2016.54	17	apoptosis	27	2016.63
3	cancer cell	67	2017.30	18	glutathione peroxidase	25	2017.28
4	GPX4	62	2017.10	19	ACSL4	24	2017.17
5	pathway	59	2017.39	20	autophagy	22	2016.95
6	inhibitor	56	2016.71	21	SLC7A11	22	2017.73
7	mechanism	53	2017.11	22	sensitivity	21	2017.71
8	iron	50	2016.52	23	neuron	20	2016.90
9	lipid peroxidation	47	2017.26	24	system x	19	2017.16
10	resistance	41	2017.49	25	*vitro*	19	2017.53
11	erastin	40	2016.22	26	lipoxygenase	18	2017.33
12	sorafenib	37	2014.54	27	glutathione	17	2017.76
13	P53	36	2016.53	28	hcc cell	16	2014.69
14	ferrostatin	33	2015.67	29	artemisinin	15	2016.00
15	reactive oxygen species	32	2016.72	30	melanoma cell	15	2019.27

AAY, average appearing year.

## Discussion

With rapid development of biomedicine, the amount of literature in biomedical domain is growing exponentially. It is reported that the volume of biomedical publications has exceeded 21 million with an annual average increase of nearly 1 million recently (45). Thus, the massive data poses a significant challenge for researchers to efficiently filter out useful information from it. Bibliometric analysis, a well-established research method using mathematical and statistical approaches, has been commonly used for revealing pervious research efforts. It is also a feasible tool to comprehensively evaluate the research advances of a certain scientific area quantitatively and qualitatively, and could predict the trends and hotspots in a specific field through information visualization (40, 46, 47). In the field of cell biology, mechanism of cell death has always been a hot topic in life science research for decades. Since the concept of ferroptosis was first proposed in 2012, endless efforts have been put into revealing its underlying molecular mechanisms and potential applications (7). In our pervious study, we have conducted a comprehensive bibliometric analysis based on the literature related to ferroptosis from 2012 to 2020 (40). Nevertheless, this study was powered based on all publications regarding ferroptosis, and top highly cited studies were not analyzed in detail. The most−cited studies in a certain field are generally considered landmarks, which have important reference value for further analysis owing to their ground−breaking contributions. In this study, we aimed to provide a comprehensive overview of the top 100 most influential studies with original data in the field of ferroptosis.

From the time distribution of these top100 articles, all of them were published between 2012 and 2020, and the years that yielded the relatively large number of high-impact articles were in 2017, 2016, and 2018. This result is consistent with our previous study. Our findings revealed that the number of publications in this domain has been continuously growing since 2012, and the past five years have witnessed an exponential growth (40). Therefore, these three years could be regarded as the inflection points for explosive growth. In addition, it must be emphasized that the total citation counts of publications in the last three years could be underestimated, considering the fact that the citations after publication usually need at least 3 years to accumulate. In order to balance the citations and temporal factors, we therefore adopted the citation density as another index to assess the average citations per year (41). The results showed that, although the total number of citations for recently published articles was lower than earlier publications, the citation densities of recently published articles were non-inferior and even higher than these early studies. It can be speculated that more and more recently published studies have chances to become highly cited articles over time.

As regards countries, the major part of the top 100 most-cited articles in the ferroptosis field was from the United States, which also owns the overwhelming number of total citation counts, indicating that the United States was the most influential country in this domain. As the largest high-tech power after the Second World War, the United States occupies a leading position in multiple global research areas (40, 48). The development of science and technology cannot be separated from the contribution from these first-class academic institutions and scholars. According to statistics, the United States owns the largest number of famous scholars and leads the tide of ferroptosis research around the world. Our result showed that nearly all of the top 10 highly cited authors were from the United States (9/11). As we all know that the concept of ferroptosis was first proposed by scholars in Columbia University (7). Columbia University and University of Pittsburgh were the institutions with the most top-cited articles among all institutions, reflecting their authority in the field of ferroptosis. In terms of the cooperation of countries or institutions, the United States collaborated most closely with China and Germany. When it comes to an institutional level, University of Pittsburgh and Guangzhou Medical University worked most closely among all institutions. This result might mainly be associated with several common research projects and Professor Tang’s team is an important nexus of them (49–52).

The top-cited studies within the research field were more likely to be published in high impact journals such as *Nature*, *Cell* and *Proceedings of the National Academy of Sciences of the United States of America*, as well as the authoritative journals of cell biology including *Cell Death and Differentiation*, *Nature Cell Biology*, *Cell Death Disease*, and *Cell Reports*, suggesting that these journals are prone to publishing original research in ferroptosis. Meanwhile, as can be seen that most of these journals have a high Impact factor value. This result also supports the well-known paradigm that high‐quality studies are often published in these journals topping the impact factor list, and in turn, maintain the high impact factor of these journals (53). Additionally, except for the field of cell biology, several studies published in these journals belonging to other fields such as materials science, nanoscience & nanotechnology, gastroenterology & hepatology, suggesting that numerous groundbreaking advances regarding ferroptosis have been made in these research directions. Take these studies published in *ACS Nano* as examples, Wang et al., reported a ferroptosis-inducing agent based on arginine-rich manganese silicate nanobubbles (AMSNs). They found that AMSNs have highly efficient glutathione depletion capabilities and subsequently leads to ferroptosis by inactivating GPX4, which provide important insights for tumor targeting with nanomedicines (54). Concurrently, Liu and colleagues from Wuhan University have constructed a ferrous-supply-regeneration SRF@Fe^III^TA nanoparticles. In brief, Fe^3+^ ion and naturally derived tannic acid formed a network-like corona onto sorafenib nanocrystal. Their results showed that SRF@Fe^III^TA can serve as an effective ferroptosis-inducing nanotherapeutic through interfering tumorous iron metabolism (55). Two other studies revealed that nanocatalysts such as PEGylated single-atom Fe-containing nanocatalysts (PSAF NCs) and Fenton-reaction-acceleratable magnetic nanoparticles could effectively trigger or accelerate tumor-specific Fenton reaction to generate abundant reactive oxygen species to induce cancer cell death (23, 56). In recent years, as nanotechnology has rapidly developed, many engineered nanomaterials that could induce ferroptosis have been developed for applications in cancer therapy. Although ferroptosis-inducing nanomedicines show some unique advantages such as increasing the active targeting to tumors, prolong the half-life in the blood, and improving antitumor ability with the synergy effect, most of the current research was only based on cell lines or animal models (57). And the biosecurity, specific mechanisms, and potential clinical application of these emerging treatments remain to be further investigated.

As shown in **Figure 7**, the citation network revealed that the study titled “Ferroptosis: An Iron-Dependent Form of Nonapoptotic Cell Death”, published in *Cell* in 2012 is the seminal article of this field (7). In this study, they have found that erastin, a small molecule compound, could selectively kill RAS-mutated cancer cells by overwhelming lipid peroxidation. This regulated cell death process depends on iron rather than other metals, and could be suppressed by iron chelators or lipophilic antioxidants like ferrostatin-1. Thus, such iron-dependent form of cell death was termed as “ferroptosis” since then. However, it is worth noting that before ferroptosis was officially named, this nonapoptotic form of cell death has already been observed *in vitro*. As early as 2003, erastin was first found through synthetic lethal high-throughput screening for tumor therapeutic drugs, and it could induce a nonapoptotic cell death process (58). Subsequently, two small molecules compounds, named RSL3 and RSL5, were found that they could increase lethality in the presence of oncogenic RAS, and activate a similar death mechanism like erastin (59). Although these two studies were not included in the top 100 articles of this field due to the setting of keywords search. They are also worth emphasizing as they have laid important groundwork for future studies. At the same time, Professor Stockwell BR and colleagues have made tremendous contributions to the establishment and progression of this field. In terms of these the top 100 articles, 19 listed his name.

Through the analysis of high-frequency terms and phrases extracted from the titles and abstracts, the top 100 most-cited articles covered a wide range of topics regarding ferroptosis. As evident from **Table 4**, in addition to term of ferroptosis, these terms or phrases including cell death, cancer cell, GPX4, pathway, inhibitor, mechanism, iron, lipid peroxidation, resistance, erastin, sorafenib, P53, reactive oxygen species, necroptosis, apoptosis, glutathione peroxidase, ACSL4, autophagy, and SLC7A11 appeared more frequently in the top 100 articles. From these terms, we could find that the current attentions of ferroptosis mainly focus on potential regulatory mechanism and pathways, key ferroptosis-related genes/molecules such as GPX4 (60, 61), P53 (62), SLC7A11 (63), ACSL4 (49, 64, 65), NRF2 (16, 66), ALOX15 (67, 68), oxidant and antioxidant system (69, 70), ferroptosis-inducing agents or nanomedicine for cancer therapy (23, 55, 56, 71), as well as the role of ferroptosis in non-neoplastic disorders such as renal failure and tubular necrosis (60, 72, 73), cardiomyopathy (74), hemorrhagic stroke (22, 75), intestinal ischemia/reperfusion (20), and neurodegenerative diseases (21, 76). Meanwhile, as shown in **Figure 9**, the combination therapeutic strategies targeting ferroptosis in radiotherapy or immunotherapy are gradually being valued (77, 78).

Regarding the potential regulatory mechanism and pathways on ferroptosis, there have been many high quality systematic and narrative reviews in **Supplementary Table1** (8, 79), and thus will not be discussed further here. In brief, the key regulatory mechanisms and pathway of ferroptosis includes iron metabolism, lipid metabolism, amino-acid metabolism, as well as other factors such as GSH-dependent pathway, transsulfuration pathway, thioredoxin, peroxiredoxin, and so on (80, 81). With increasing ferroptotic mechanisms were clarified and ferroptosis inducers/inhibitors were found, there is increasing number of studies focusing on the discovery of ferroptosis-inducing agents such as small molecules and nanomaterials to eradicate malignancies (82–84). Ferroptosis inducers such as erastin, could be used with various chemotherapeutic agents including temozolomide, doxorubicin, cisplatin, and cytarabine in different type of cancers (66, 85). However, ferroptosis is also considered to be related to the onset of various diseases (74, 86). Thus, controlling the dosage of ferroptosis inducers and improving the specificity are essential for reducing the adverse events on normal tissues. And it is also of great clinical significance to clarify the key mechanism regulating ferroptotic sensitivity to tumor cells and avoiding the escape of malignancies from anticancer modalities.

Additionally, an increasing number of studies have confirmed that ferroptosis could interact with immune cells and enhance the immunogenicity of cancer cells. A study by Wang and colleagues found that CD8^+^ T cells activated by immune checkpoint blockade were able to promote ferroptosis-specific lipid peroxidation in tumor cells by secreting interferon gamma (IFNγ). Mechanistically, IFNγ significantly downregulated the expression of SLC3A2 and SLC7A11, and then impaired the uptake of cystine by tumor cells, which resulted in enhanced lipid peroxidation and ferroptosis (77). Meanwhile, ferroptosis can expose tumor antigens and improve the immunogenicity of tumor microenvironment and thereby promoting T-cell activation and facilitating antitumor T-cell immune response (87, 88). A study conducted by Lang et al. found that ferroptosis was a novel point of synergy between radiotherapy and immunotherapy. Immunotherapy sensitizes tumors to radiotherapy by promoting tumor-cell ferroptosis and SLC7A11 was a critical regulator in this process (78). Consequently, ferroptosis plays an essential role in the regulation of T-cell-mediated cellular immunity. Induction of ferroptosis through iron deposition-based combination strategies with direct or indirect ferroptosis inducers emerges to be promising therapeutic approaches to improve anti-PD-1/PD-L1 immunotherapy.

## Limitations

This study has several limitations of note. First, we only used WoSCC database as data source, which may miss several related publications in other databases such as Scopus and Pubmed. However, different databases have different ways to count citations, this may be inappropriate to merge data from different databases. Among these databases, WoSCC is the most commonly used for analyzing the highly cited articles in a certain field (34, 35, 38). And it is generally thought that WoSCC is able to represent the condition of most publications in a field and is a reliable source of international peer-reviewed publications (89–91). Second, it should be noted that the frequency of citations in earlier studies should be higher than the recently published ones owing to the time factor, though the academic impact of former ones may be not really stronger than that of later ones (92). Thus, some breakthrough works published recently might be excluded due to lack of sufficient time to accumulate citations. Third, it remains controversial whether citations counts could reflect academic influence, though it is a widely used reference index (93). We are in agreement with the viewpoint that citations might not be fully representative of real academic values and the impact of one study should be evaluated comprehensively. In spite of this, the results of this study still could answer several important questions.

## Conclusion

Overall, based on bibliometric analysis of the publications over the past decade, the top 100 most influential articles regarding ferroptosis were identified to provide a comprehensive and quantitative analysis of the key contributions made to drive the evolution of this field. Moreover, the current hotspots were also identified to provide useful insights for scholars. Since the ferroptosis concept was proposed in 2012, it has been noted that the ferroptosis related domain is developing tremendously. The USA could be viewed as the dominant country in terms of the number of high-impact articles, world-class academic institutions, and leading scientists in this research field. Currently, research in the field of ferroptosis is mainly focused on potential regulatory mechanism and pathways, key ferroptosis-related genes/molecules, oxidant and antioxidant system, ferroptosis-inducing agents or nanomedicine for cancer therapy, as well as combination therapeutic strategies. Continued in-depth studies in this area will contribute to a better understanding of the molecular pathophysiological mechanisms of multiple diseases and help to discover more potential therapeutic targets.

## Author contributions

KC, ZSh, and HW designed the study. KC, QG, ZSh, and WY collected the data. KC, ZSu, YZ, WY, XY, and HW analyzed the data and drafted the manuscript. KC, QG, ZSh, YZ, WY, SunZ, XY, and HW revised and approved the final version of the manuscript. All authors read and approved the submitted version.

## Funding

This research was funded by the Tianjin Health Science and Technology Project (Grant No. QN20016).

## Acknowledgments

The authors thank Professor Xiuhua Yao from Tianjin huanhu hospital and “home-for-researchers (www.home-for-researchers.com)” for their effort in polishing the English content of this manuscript.

## Conflict of interest

The authors declare that the research was conducted in the absence of any commercial or financial relationships that could be construed as a potential conflict of interest.

## Publisher’s note

All claims expressed in this article are solely those of the authors and do not necessarily represent those of their affiliated organizations, or those of the publisher, the editors and the reviewers. Any product that may be evaluated in this article, or claim that may be made by its manufacturer, is not guaranteed or endorsed by the publisher.
