# Supplementary material for: Frontiers of ferroptosis research: An analysis from the top 100 most influential articles in the field
Source: Front Oncol. 2022 Aug 11;12:948389. doi: 10.3389/fonc.2022.948389 (PMC9403769; doi:10.3389/fonc.2022.948389)
Supplement: Supplementary file 1 [file DataSheet_1.docx]

Supplementary Material

# Supplementary Figure





**SUPPLEMENTARY FIGURE 1. Citation densities of each article**

# Supplementary Table

**SUPPLEMENTARY TABLE1. Top 50** **highly cited review articles on ferroptosis ranked according to their total citations counts**

| Ranking | Title | Total citations | AC per year | Journal | First Author | Published  year |
| --- | --- | --- | --- | --- | --- | --- |
| 1 | Ferroptosis: A Regulated Cell Death Nexus Linking Metabolism, Redox Biology, and Disease | 1761 | 293.5 | *CELL* | Stockwell, Brent R | 2017 |
| 2 | Molecular mechanisms of cell death: recommendations of the Nomenclature Committee on Cell Death 2018 | 1715 | 343 | *CELL DEATH AND DIFFERENTIATION* | Galluzzi, Lorenzo | 2018 |
| 3 | Ferroptosis: process and function | 1039 | 148.43 | *CELL DEATH AND DIFFERENTIATION* | Xie, Y | 2016 |
| 4 | The role of iron and reactive oxygen species in cell death | 1009 | 112.11 | *NATURE CHEMICAL BIOLOGY* | Dixon, Scott J | 2014 |
| 5 | Regulated necrosis: the expanding network of non-apoptotic cell death pathways | 980 | 108.89 | *NATURE REVIEWS MOLECULAR CELL BIOLOGY* | Vanden Berghe, Tom | 2014 |
| 6 | Ferrootosis: Death by Lipid Peroxidation | 848 | 121.14 | *TRENDS IN CELL BIOLOGY* | Yang, Wan Seok | 2016 |
| 7 | Lipid peroxidation in cell death | 593 | 98.83 | *BIOCHEMICAL AND BIOPHYSICAL RESEARCH COMMUNICATIONS* | Gaschler, Michael M | 2017 |
| 8 | The molecular machinery of regulated cell death | 514 | 128.5 | *CELL RESEARCH* | Tang, Daolin | 2019 |
| 9 | Targeting Ferroptosis to Iron Out Cancer | 498 | 124.5 | *CANCER CELL* | Hassannia, Behrouz | 2019 |
| 10 | Mechanisms of ferroptosis | 494 | 70.57 | *CELLULAR AND MOLECULAR LIFE SCIENCES* | Cao, Jennifer Yinuo | 2016 |
| 11 | Reactive oxygen species and cancer paradox: To promote or to suppress? | 448 | 74.67 | *FREE RADICAL BIOLOGY AND MEDICINE* | Galadari, Sehamuddin | 2017 |
| 12 | Ferroptosis: past, present and future | 426 | 142 | *CELL DEATH & DISEASE* | Li, Jie | 2020 |
| 13 | Mitochondria as multifaceted regulators of cell death | 397 | 132.33 | *NATURE REVIEWS MOLECULAR CELL BIOLOGY* | Bock, Florian J | 2020 |
| 14 | Recent Progress in Ferroptosis Inducers for Cancer Therapy | 332 | 83 | *ADVANCED MATERIALS* | Liang, Chen | 2019 |
| 15 | NEURONAL CELL DEATH | 329 | 65.8 | *PHYSIOLOGICAL REVIEWS* | Fricker, Michael | 2018 |
| 16 | Ferroptosis at the crossroads of cancer-acquired drug resistance and immune evasion | 320 | 80 | *NATURE REVIEWS CANCER* | Angeli, Jose Pedro Friedmann | 2019 |
| 17 | Regulated necrosis: disease relevance and therapeutic opportunities | 309 | 44.14 | *NATURE REVIEWS DRUG DISCOVERY* | Conrad, Marcus | 2016 |
| 18 | Regulators of Iron Homeostasis: New Players in Metabolism, Cell Death, and Disease | 299 | 42.71 | *TRENDS IN BIOCHEMICAL SCIENCES* | Bogdan, Alexander R | 2016 |
| 19 | Reactive Oxygen Species-Induced Lipid Peroxidation in Apoptosis, Autophagy, and Ferroptosis | 285 | 71.25 | *OXIDATIVE MEDICINE AND CELLULAR LONGEVITY* | Su, Lian-Jiu | 2019 |
| 20 | Emerging Strategies of Cancer Therapy Based on Ferroptosis | 279 | 55.8 | *ADVANCED MATERIALS* | Shen, Zheyu | 2018 |
| 21 | Ferroptosis, a new form of cell death: opportunities and challenges in cancer | 274 | 68.5 | *JOURNAL OF HEMATOLOGY & ONCOLOGY* | Mou, Yanhua | 2019 |
| 22 | NRF2 plays a critical role in mitigating lipid peroxidation and ferroptosis | 271 | 67.75 | *REDOX BIOLOGY* | Dodson, Matthew | 2019 |
| 23 | Regulated Cell Death in AKI | 271 | 30.11 | *JOURNAL OF THE AMERICAN SOCIETY OF NEPHROLOGY* | Linkermann, Andreas | 2014 |
| 24 | Ferroptosis: Role of lipid peroxidation, iron and ferritinophagy | 256 | 42.67 | *BIOCHIMICA ET BIOPHYSICA ACTA-GENERAL SUBJECTS* | Latunde-Dada, Gladys O | 2017 |
| 25 | The development of the concept of ferroptosis | 235 | 58.75 | *FREE RADICAL BIOLOGY AND MEDICINE* | Hirschhorn, Tal | 2019 |
| 26 | Induction of reactive oxygen species: an emerging approach for cancer therapy | 230 | 38.33 | *APOPTOSIS* | Zou, Zhengzhi | 2017 |
| 27 | Ferroptosis: mechanisms, biology and role in disease | 229 | 114.5 | *NATURE REVIEWS MOLECULAR CELL BIOLOGY* | Jiang, Xuejun | 2021 |
| 28 | Ferroptosis: machinery and regulation | 213 | 71 | *AUTOPHAGY* | Chen, Xin | 2021 |
| 29 | Ferroptosis, a new form of cell death, and its relationships with tumourous diseases | 213 | 35.5 | *JOURNAL OF CELLULAR AND MOLECULAR MEDICINE* | Yu, Haitao | 2017 |
| 30 | Unsolved mysteries: How does lipid peroxidation cause ferroptosis? | 211 | 42.2 | *PLOS BIOLOGY* | Feng, Huizhong | 2018 |
| 31 | Role of GPX4 in ferroptosis and its pharmacological implication | 210 | 52.5 | *FREE RADICAL BIOLOGY AND MEDICINE* | Seibt, Tobias M | 2019 |
| 32 | Ferroptosis Inhibition: Mechanisms and Opportunities | 206 | 34.33 | *TRENDS IN PHARMACOLOGICAL SCIENCES* | Angeli, Jose Pedro Friedmann | 2017 |
| 33 | Iron and cancer: recent insights | 203 | 29 | *COOLEY'S ANEMIA* | Manz, David H | 2016 |
| 34 | Lipid Peroxidation-Dependent Cell Death Regulated by GPx4 and Ferroptosis | 199 | 33.17 | *APOPTOTIC AND NON-APOPTOTIC CELL DEATH* | Imai, Hirotaka | 2017 |
| 35 | Amino acid transporter SLC7A11/xCT at the crossroads of regulating redox homeostasis and nutrient dependency of cancer | 192 | 38.4 | *CANCER COMMUNICATIONS* | Koppula, Pranavi | 2018 |
| 36 | Ferroptosis: molecular mechanisms and health implications | 191 | 63.67 | *CELL RESEARCH* | Tang, Daolin | 2021 |
| 37 | FUNDAMENTAL MECHANISMS OF REGULATED CELL DEATH AND IMPLICATIONS FOR HEART DISEASE | 190 | 47.5 | *PHYSIOLOGICAL REVIEWS* | Del Re, Dominic P | 2019 |
| 38 | Broadening horizons: the role of ferroptosis in cancer | 187 | 93.5 | *NATURE REVIEWS CLINICAL ONCOLOGY* | Chen, Xin | 2021 |
| 39 | Ferroptosis is a type of autophagy-dependent cell death | 186 | 62 | *SEMINARS IN CANCER BIOLOGY* | Zhou, Borong | 2020 |
| 40 | The chemical basis of ferroptosis | 179 | 44.75 | *NATURE CHEMICAL BIOLOGY* | Conrad, Marcus | 2019 |
| 41 | The Roles of NRF2 in Modulating Cellular Iron Homeostasis | 172 | 34.4 | *ANTIOXIDANTS & REDOX SIGNALING* | Kerins, Michael John | 2018 |
| 42 | The Hallmarks of Ferroptosis | 171 | 42.75 | *ANNUAL REVIEW OF CANCER BIOLOGY, VOL 3* | Dixon, Scott J | 2019 |
| 43 | Striking while the iron is hot: Iron metabolism and ferroptosis in neurodegeneration | 165 | 41.25 | *FREE RADICAL BIOLOGY AND MEDICINE* | Masaldan, Shashank | 2019 |
| 44 | Regulation of lipid peroxidation and ferroptosis in diverse species | 163 | 32.6 | *GENES & DEVELOPMENT* | Conrad, Marcus | 2018 |
| 45 | The Coming Decade of Cell Death Research: Five Riddles | 159 | 39.75 | *CELL* | Green, Douglas R. | 2019 |
| 46 | Ferroptosis and cell death mechanisms in Parkinson's disease | 157 | 26.17 | *NEUROCHEMISTRY INTERNATIONAL* | Guiney, Stephanie J | 2017 |
| 47 | Iron and Ferroptosis: A Still Ill-Defined Liaison | 154 | 25.67 | *IUBMB LIFE* | Doll, Sebastian | 2017 |
| 48 | GPx4, Lipid Peroxidation, and Cell Death: Discoveries, Rediscoveries, and Open Issues | 153 | 30.6 | *ANTIOXIDANTS & REDOX SIGNALING* | Maiorino, Matilde | 2018 |
| 49 | Ferroptosis, necroptosis, and pyroptosis in anticancer immunity | 150 | 50 | *JOURNAL OF HEMATOLOGY & ONCOLOGY* | Tang, Rong | 2020 |
| 50 | Targeting Nrf2 to Suppress Ferroptosis and Mitochondrial Dysfunction in Neurodegeneration | 149 | 29.8 | *FRONTIERS IN NEUROSCIENCE* | Abdalkader, Moataz | 2018 |

AC, average citation
